# The influence of deliberate practice on musical achievement: a meta-analysis

**DOI:** 10.3389/fpsyg.2014.00646

**Published:** 2014-06-25

**Authors:** Friedrich Platz, Reinhard Kopiez, Andreas C. Lehmann, Anna Wolf

**Affiliations:** ^1^University of Music and Performing ArtsStuttgart, Germany; ^2^Hanover Music Lab, Hanover University of Music, Drama and MediaHanover, Germany; ^3^University of MusicWürzburg, Germany

**Keywords:** deliberate practice, music, sight-reading, meta-analysis, expert performance

## Abstract

Deliberate practice (DP) is a task-specific structured training activity that plays a key role in understanding skill acquisition and explaining individual differences in expert performance. Relevant activities that qualify as DP have to be identified in every domain. For example, for training in classical music, solitary practice is a typical training activity during skill acquisition. To date, no meta-analysis on the quantifiable effect size of deliberate practice on attained performance in music has been conducted. Yet the identification of a quantifiable effect size could be relevant for the current discussion on the role of various factors on individual difference in musical achievement. Furthermore, a research synthesis might enable new computational approaches to musical development. Here we present the first meta-analysis on the role of deliberate practice in the domain of musical performance. A final sample size of 13 studies (total *N* = 788) was carefully extracted to satisfy the following criteria: reported durations of task-specific accumulated practice as predictor variables and objectively assessed musical achievement as the target variable. We identified an aggregated effect size of *r*_*c*_ = 0.61; 95% CI [0.54, 0.67] for the relationship between task-relevant practice (which by definition includes DP) and musical achievement. Our results corroborate the central role of long-term (deliberate) practice for explaining expert performance in music.

## Introduction

Current research on individual differences in the domain of music is surrounded by controversial discussions: On the one hand, exceptional achievement is explained within the expert-performance framework with an emphasis on the role of structured training as the key variable; on the other hand, researchers working in the individual differences framework argue that (possibly innate) abilities and other influential variables (e.g., working memory) may explain observable inter-individual differences (see Ericsson, 2014 for a detailed discussion). The expert-performance approach is represented by studies by Ericsson and coworkers (e.g., Ericsson et al., 1993) who assume that engaging in relevant domain-related activities, especially deliberate practice (DP), is necessary and moderates attained level of performance. Deliberate practice is qualitatively different from work and play and “includes activities that have been specially designed to improve the current level of performance” (p. 368). In a more comprehensive and detailed definition, Ericsson and Lehmann (1999) refer to DP as a

“Structured activity, often designed by teachers or coaches with the explicit goal of increasing an individual's current level of performance. (···) it requires the generation of specific goals for improvement and the monitoring of various aspects of performance. Furthermore, deliberate practice involves trying to exceed one's previous limit, which requires full concentration and effort.” (p. 695)

In other words, we have to distinguish between mere experience (as a non-directed activity) and deliberate practice. An individual's involvement with a new domain entails the accumulation of experience, which may include practice components and lead to initially acceptable levels of performance. However, only the conscious use of strategies along with the desire to improve will result in superior expert performance (Ericsson, 2006). Note that in most studies DP is only indirectly estimated using durations of task-relevant training activities that also include an unspecified proportion of non-deliberate practice components. The unreflected use of the “accumulated deliberate practice” concept to denote durations of accumulated time spent in training activities is therefore misleading, because the measured durations might theoretically underestimate the true effect of deliberate practice on attained performance. In the context of classical music performance, the task-relevant activity can often consist of some type of solitary practice (e.g., studying repertoire or practicing scales) or the execution of a particular activity in a rehearsal or training context (e.g., sight-reading at the piano while coaching a soloist; receiving lessons). The theoretical framework for the explanation of expert and exceptional achievement has been validated in various domains and is widely accepted nowadays (Ericsson, 1996), as evidenced by the extremely high citation frequencies of key publications in this area. For example, according to Google Scholar, the study by Ericsson et al. (1993) has been cited more than 4000 times in the 20 years since its publication. As an internationally known proponent of research on giftedness, Ziegler (2009) concludes that even modern conceptions of giftedness research have integrated the perspective of expertise theory. However, controversial discussions persist (see Detterman, 2014).

In contrast, researchers relying more on talent-based approaches maintain that DP might not explain individual differences in performance sufficiently and emphasize innate variables as the explanation for outstanding musical achievement, such as working memory capacity (Vandervert, 2009; Meinz and Hambrick, 2010), handedness (Kopiez et al., 2006, 2010, 2012), sensorimotor speed (Kopiez and Lee, 2006, 2008), psychometric intelligence (Ullén et al., 2008), intrinsic motivation (Winner, 1996), unique type of representations (Shavinina, 2009), or verbal memory (Brandler and Rammsayer, 2003). According to Ericsson (2014), the predictive power of additional factors, such as general cognitive abilities, is usually of small to medium size and diminishes as the level of expertise increases.

Although expertise theory provides convincing arguments for the importance of structured training on expert skill acquisition and achievement, no comprehensive quantification for the influence of DP on musical achievement has been presented so far. A first and highly commendable attempt to estimate the “true” (population) effect of DP via estimates of durations of accumulated practice on musical achievement was published by Hambrick et al. (2014) who identified a sample of eight studies for their review. However, their methodology, assumptions, and use of the term DP raise some issues that have to be resolved. These open questions and concerns spawned our initial motivation for the present meta-analysis.

### Reanalysis of data presented in Hambrick et al. (2014)

First, we carefully studied the publication by Hambrick et al. (2014), (Table 1). Using Table 3 of their paper, we extracted the correlations between training data and measures of music performance and entered these data into a meta-analysis software (Comprehensive Meta-Analysis, see Borenstein, 2010). This analysis brought to light an aggregated efffect size value of *r* = 0.44 for the influence of training data on musical performance (see Table 1, for details). According to Cohen's (1988) benchmarks, this corresponds to a large overall effect (see also Ellis, 2010, p. 41). Unlike Hambrick et al. (2014), we did not use the correlation values corrected for measurement error variance (attenuation correction) in the present paper because their correction of confidence intervals relied on the biased Fisher's *z* transformation (see Hunter and Schmidt, 2004, Ch. 5) and not on the corrected sampling error variance for each individual correlation as suggested by Hunter and Schmidt (2004, Ch. 3). Therefore, to allow for later comparisons, we decided to use the uncorrected (attenuated) correlation as the basis for our analysis of heterogeneity.

**Table 1:** **Aggregation of data from Table 3 in Hambrick et al. (2014) for the reanalysis of effect sizes regarding the influence of deliberate practice on music performance**.

**Study**	***N***	**Variance**	***r* (95% CI)**	**Relative weight [%]**
Lehmann and Ericsson, 1996	16	0.07	0.36 (−0.17, 0.73)	2.15
Meinz, 2000	107	0.01	0.41 (−0.24, 0.56)	17.22
Tuffiash, 2002	135	0.01	0.58 (−0.46, 0.68)	21.85
Kopiez and Lee, 2008	52	0.02	0.25 (−0.03, 0.49)	8.11
Ruthsatz et al., 2008—study 1	178	0.01	0.34 (−0.20, 0.46)	28.97
Ruthsatz et al., 2008—study 2A	64	0.01	0.31 (−0.07, 0.52)	10.10
Ruthsatz et al., 2008—study 2B	19	0.06	0.54 (−0.11, 0.80)	2.65
Meinz and Hambrick, 2010	57	0.02	0.67 (−0.50, 0.79)	8.94
**MEAN AGGREGATED EFFECT SIZE**				
Fixed effect model			0.44 (−0.37, 0.50)	
Random effects model			0.44 (−0.33, 0.55)	

*Aggregation of studies shows a large (*I*^2^ = 60.3%) and significant heterogeneity (Q(7) = 17.7, p < 0.02)*.

The effect size, however, is not the only relevant parameter in a meta-analysis, and it should be examined in the light of a possible publication bias. To test for the strength of the resulting effect size estimate, we conducted a test for heterogeneity for the underlying sample of studies. Following Deeks et al. (2008), the *I*^2^ value describes the percentage of variance in effect size estimates that can be attributed to heterogeneity rather than to sampling error. The *I*^2^ value of 60.3 obtained for the Hambrick et al. (2014) sample of studies implied that it “may represent substantial heterogeneity” (Deeks et al., 2008, p. 278). The main reason for possible heterogeneity, in our opinion, could be a less selective inclusion with resulting inconsistent predictor and target variables. For example, in their study on the acquistion of expertise in musicians, Ruthsatz et al. (2008) used inconsistent (non-standardized) indicators for the estimation of musical achievement that made it difficult to compare the observed differences in performance: In Study 1, the band director's audition scores for each of the high school band members were ranked and used as individual indicators of musical achievement; in Study 2A, audition scores from the admission exam were used as the outcome variable; and in Study 2B, a music faculty member rated the students' general musical achievement. In no instance was a standardized performance task used as the target variable. Unfortunately, no information was reported on the rating reliabilities.

Although our reanalysis of Hambrick et al.'s(2014) review confirmed a large effect size for the relation between training data and musical achievement, this finding still underestimates the “true” value. In order to arrive at a convincing effect size for deliberate practice in the domain of music we also aggregated studies, but invested great effort in the selection of studies for our meta-analysis. As will be shown below, our meta-analysis was not affected by potential publication bias and heterogeneity. We also applied transparent and consistent criteria for study selection as this is one of the most important prerequisites for the aggregation of studies.

### Choice of method

Two methods are available to evaluate past research: (a) a narrative and systematic review and (b) a meta-analysis. The narrative reviewer uses published studies, reports other authors' results in his or her own words and draws conclusions (Ellis, 2010, p. 89). A systematic review is also sometimes referred to as a “qualitative review” or “thematic synthesis” (Booth et al., 2012) and necessitates a comprehensive search of the literature. The disadvantage of this approach is that it depends on the availability of results published in established journals and tends to show a publication bias toward the Type I error (false positive). The reason for this is that journals prefer to publish studies with significant results, and negative findings or null results have a lower probability of publication (Masicampo and Lalande, 2012). In the field of music, narrative reviews on the influence of DP on musical achievement play an important role and have been conducted in the last two decades (Lehmann, 1997, 2005; Howe et al., 1998; Sloboda, 2000; Krampe and Charness, 2006; Lehmann and Gruber, 2006; Gruber and Lehmann, 2008; Campitelli and Gobet, 2011; Hambrick and Meinz, 2011; Nandagopal and Ericsson, 2012; Ericsson, 2014).

The other approach is that of a meta-analysis. Here, studies are included following “pre-specified eligibility criteria in order to answer a specific research question” (Higgins and Green, 2008, p. 6). Within the meta-analytic approach, studies' effect sizes have to be weighted before they are aggregated. Every study's effect size weight then reflects its degree of precision as a function of sample size (Ellis, 2010). Consequently, studies with smaller sample sizes, particularly in combination with larger variation, will result in smaller weights compared to studies with larger sample sizes and more narrow variation. These weights of the individual studies then function as estimators of precision. If these weights differ markedly from each other, statistical heterogeneity is present. The final result of a meta-analysis is the weighted mean effect size across all studies included. Compared to an individual study's effect size, this weighted mean effect size represents a more precise point estimate as well as an interval estimate surrounding the effect size in the population (Ellis, 2010, p. 95). Moreover, a meta-analysis generally increases statistical power by reducing the standard error of the weighted average effect size (Cohn and Becker, 2003). Researchers who use meta-analysis techniques have two goals: First, they want to arrive at an interval of effect size estimation in a population based on aggregated effect sizes of individual studies; second, they want to give an evidence-based answer to those questions that reviews or replication studies cannot give in part due to their arbitrary collection of significant and insignificant results.

Despite the fact that meta-analyses have been shown to be an important constituent for the production of “verified knowledge” (Kopiez, 2012), they have only recently been applied to various topics in music psychology (e.g., Chabris, 1999; Hetland, 2000; Pietschnig et al., 2010; Kämpfe et al., 2011; Platz and Kopiez, 2012; Mishra, 2014). To date, there has been no formal meta-analysis concerning the influence of DP on attained music performance.

### Goal of the present study

The aim of our study was two-fold: First, by means of a systematic literature review we wanted to identify all relevant publications that might help us answer the question of how strongly task-specific practice influences attained music performance. Second, we wanted to quantify the effect of DP on music performance in terms of an objectively computed effect size. This effect size is an important component for the development of a comprehensive model for the explanation of individual differences in the domain of music. Although this meta-analysis is supposed to reveal the “true” effect size of deliberate practice on musical achievement, for theoretical reasons it is possible that it is still underestimating the upper bound of deliberate practice (see Future Perspectives).

## Materials and methods

The study was conducted in three steps: First, to arrive at a relevant sample of selected studies, we conducted a systematic review (Cooper et al., 2009) that helped to control for publication bias (Rothstein et al., 2005). In the second step, we identified each study's predictor and outcome variable in line with Ericsson (2014), and we identified all artifactual confounds that might attenuate the studies' outcome measures (Hunter and Schmidt, 2004, p. 35). Third, we carried out a meta-analysis of individually corrected (disattenuated) correlations as well as a quantification of its variance (Hunter and Schmidt, 2004; Schmidt and Le, 2005) to obtain the true mean score correlation (ρ) between music-related practice and musical achievement.

### Sample of selected studies

**Figure 1:**
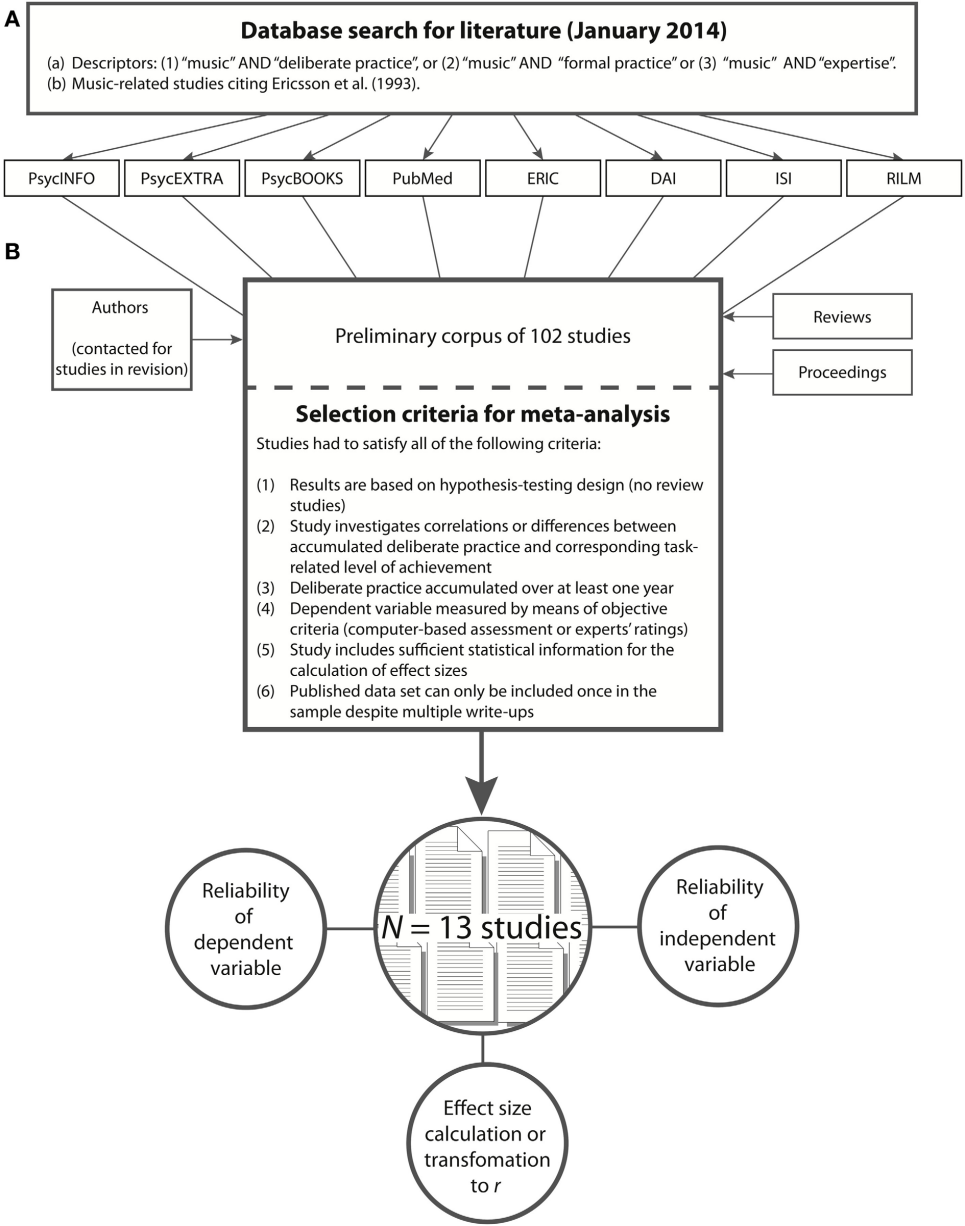
**Arriving at a study sample for the meta-analysis**. In the first step **(A)**, a search for literature was based on selected descriptors applied to eight data bases. This resulted in a preliminary corpus of 102 studies. In the second step **(B)**, studies were evaluated and selected for meta-analysis according to seven criteria. *N* = 13 studies matched all criteria and were included into the meta-analysis.

Our sample of selected studies for the subsequent meta-analysis was the outcome of a systematic literature search which had led to a preliminary corpus of selected studies (see Figure 1A). Due to a wide variety of methodological approaches, and for the purpose of later generalizability of our meta-analytical results, we decided to select only studies with comparable experimental designs. Therefore, in the next step of generating a sample, we excluded all studies from the preliminary corpus that did not meet all of our selection criteria (see Figure 1B). Consequently, our preliminary corpus of *n* = 102 studies dwindled to the final sample of *n* = 13 studies which served as input for the meta-analysis.

### Literature search

The acquisition of studies for our systematic review derived from (a) the search for relevant databases of scientific literature, (b) queries of conference proceedings, and (c) personal communications with experts in the field of music education or musical development. First, a database backward and forward search for literature was conducted in January 2014 (Figure 1A). To control for publication bias (see Rothstein et al., 2005), we considered a large variety of databases for our literature search: peer-reviewed studies in the field of medical and neuroscientific (PubMed), psychological (PsycINFO), educational (ERIC), social (ISI), and musicological research (RILM). To avoid an overestimation of the effect size due to possibly unpublished results (Rosenthal, 1979), so-called “gray literature” (Rothstein and Hopewell, 2009) with often non-significant study results, we also searched doctoral dissertations (DAI), proceedings or newspaper articles (PsycEXTRA) as well as book chapters containing psychological study results (PsycBOOKS).

Studies were excluded from the preliminary corpus if they did not conform with at least one of the following three descriptors (Figure 1A): (1) “music” AND “deliberate practice,” (2) “music” AND “formal practice,” (3) “music” AND “expertise.” In addition, we included in the preliminary corpus those music-related studies which cited Ericsson et al.'s (1993) first extensive review of skill acquisition research. Finally, authors who had conducted experimental studies on predictors of music achievement were contacted and queried for currently unpublished correlational data involving music-related deliberate practice and musical achievement. In total, our initial literature search resulted in a preliminary corpus of 102 studies (Figure 1A).

### Criteria-related literature selection

While Hambrick et al. (2014) performed a more intuitive search, resulting in a significant heterogeneity of the study sample, the aim of our method was to arrive at a homogenous sample of pertinent studies. To this end, we selected studies based on objective criteria which we derived from the theoretical framework of expert performance according to Ericsson et al. (1993). Thus, studies were successively removed from the preliminary corpus of studies if they did not meet all the criteria shown in Figure 1B. As a result of our study selection (see Table 2), we identified studies which met the following 6 criteria: (1) they followed a hypothesis-testing design; (2) they contained a correlation between accumulated deliberate practice and a *corresponding* task-related level of musical achievement; (3) the amount of relevant practice had to be accrued across at least 1 year, (4) musical performance had to be measured by means of objective criteria such as a computer-based assessment (e.g., scale analysis by Jabusch et al., 2004) or expert evaluation based on psychometric scales (e.g., Hallam, 1998). (5) Furthermore, studies were excluded if they did not contain sufficient statistical information for effect size calculation or estimation. (6) Finally, in the case of duplicate publication of data (as happens when original articles are also published in chapter form), study results were considered only once for effect size aggregation in the meta-analysis.

**Table 2:** **Studies, included in meta-analysis**.

**ID**	**Study**	**Comments**
Kornicke, 1992	Kornicke, L. E. (1992). *An exploratory study of individual difference variables in piano sight-reading achievement* (Doctoral Dissertation, Indiana University, Ann Arbor, USA). Available from ProQuest Dissertations and Theses database. (UMI No. 9301458).	
Ericsson et al., 1993—Study II	Ericsson, K. A., Krampe, R. T., and Tesch-Römer, C. (1993). The role of deliberate practice in the acquisition of expert performance. *Psychological Review 100*, 363–406.	Two studies reported; only data of study II was considered.
Lehmann and Ericsson, 1996	Lehmann, A. C., and Ericsson, K. A. (1996). Performance without preparation: structure and acquisition of expert sight-reading and accompanying performance. *Psychomusicology 15*, 1–29.	
Krampe and Ericsson, 1996—Study I	Krampe, R. T., and Ericsson, K. A. (1996). Maintaining excellence: deliberate practice and elite performance in young and older pianists. *Journal of Experimental Psychology: General 125*, 331–359.	Two studies reported; only data of study I was considered.
Hallam, 1998	Hallam, S. (1998). The predictors of achievement and dropout in instrumental tuition. *Psychology of Music 26*, 116–132.	
Meinz, 2000	Meinz, E. J. (2000). Experience-based attenuation of age-related differences in music cognition tasks. *Psychology and Aging 15*, 297–312.	
Tuffiash, 2002	Tuffiash, M. (2002). *Predicting individual differences in piano sight-reading skill: practice, performance, and instruction*. Unpublished master's thesis, Florida State University, Tallahassee, FL.	
McPherson, 2005	McPherson, G. E. (2005). From child to musician: skill development during the beginning stages of learning an instrument. *Psychology of Music 33*, 5–35.	Author contacted for data.
Jabusch et al., 2007	Jabusch, H.-C., Yong, R., and Altenmüller, E. (22–23 Nov. 2007). *Biographical predictors of music-related motor skills in children pianists*. Paper presented at the International Symposium on Performance Science, Porto.	
Kopiez and Lee, 2008	Kopiez, R., and Lee, J. I. (2008). Towards a general model of skills involved in sight reading music. *Music Education Research 10*, 41–62.	
Jabusch et al., 2009	Jabusch, H. C., Alpers, H., Kopiez, R., Vauth, H., and Altenmüller, E. (2009). The influence of practice on the development of motor skills in pianists: a longitudinal study in a selected motor task. *Human Movement Science 28*, 74–84.	
Meinz and Hambrick, 2010	Meinz, E. J., and Hambrick, D. Z. (2010). Deliberate practice is necessary but not sufficient to explain individual differences in piano sight-reading skill: the role of working memory capacity. *Psychological Science 21*, 914–919.	
Kopiez et al., 2012—Study II	Kopiez, R., Jabusch, H.-C., Galley, N., Homann, J.-C., Lehmann, A. C., and Altenmüller, E. (2012). No disadvantage for left-handed musicians: the relationship between handedness, perceived constraints and performance-related skills in string players and pianists. *Psychology of Music 40*, 357–384.	Two studies reported; only data of study II was considered.

Following our selection criteria *n* = 89 studies had to be excluded from our preliminary corpus. Our final sample size was thus *n* = 13 studies, comprising results from peer-reviewed studies as well as “gray” literature from 1992 to 2012 (see Table 2). For comparison, Hambrick et al.'s (2014) sample size of studies included in his review was *n* = 8.

### Procedure

According to Hunter and Schmidt (2004, p. 33), the aim of a psychometric meta-analysis is two-fold: namely, to uncover the variance of observed effect sizes (*s*^2^_*r*_)—in our study, this was the variance of observed correlations between the task-related practice (predictor) and musical achievement (outcome variable)—and to estimate the supposedly “true” effect size distribution in the population (σ^2^_ρ_). The use of the term “psychometric” refers to the idea in classical testing theory (Gulliksen, 1950) that every observed correlation is subject to an attenuation due to the imperfect measurement of variables, sampling error, and further artifacts (for an overview see Hunter and Schmidt, 2004, p. 35). If the influence of all such artifactual influences on an observed correlation are known (*r_o_*), each study's correlation can be corrected first for its individual attenuation bias (*r_c_*). In a subsequent step, the population variance of the “true” correlation (σ^2^_ρ_) is estimated by subtracting the observed variance of corrected correlations (*s^2^_r_c__*) from the observed variance attributable to all attenuating factors (*s^2^_e_c__*). In the case of a perfect concordance between the observed variance of corrected correlations (*s^2^_r_c__*) and the observed variance attributable to all artifacts (*s^2^_e_c__*), there is no population variance left to be explained (σ^2^_ρ_ = 0). Then all studies' effect sizes in the meta-analysis are homogenous and assumed to derive from one single population effect (Hunter and Schmidt, 2004, p. 202). Therefore, we will first identify each study's theoretically appropriate predictor and outcome variable as well as reliability information for both variables in order to calculate effect size and estimate artifactual influence.

### Identification of predictors and outcome variables

Although accumulated deliberate practice on an instrument has been identified as a generally important biographical predictor in the acquisition of expert performance (Ericsson et al., 1993), it is sometimes erroneously considered a catch-all predictor for achievement in music-specific tasks. However, as Ericsson clearly states, “it is not the total number of hours of practice that matter, but a *particular type of practice* [emphasis by the third author, AL] that predicts the difference between elite and sub-elite athletes” (Ericsson, 2014, p. 94). For example, according to Lehmann and Ericsson (1996) as well as Kopiez and Lee (2006, 2008), sight-reading performance as a domain-specific task of musical achievement should be less well predicted by accumulated generic deliberate practice in piano playing (i.e., solitary practice) than by the accumulated amount of task-specific deliberate practice in the field of accompanying and sight-reading. Therefore—and in contrast to Hambrick et al.'s (2014) procedure—for each study we identified the most corresponding predictor variable. For example, the researcher might have summed up the number of pieces sight-read (Kornicke, 1992, p. 133), determined the size of the accompanying repertoire (Lehmann and Ericsson, 1996, p. 29), counted the number of accompanying performances (Meinz, 2000, p. 301), reported cumulated piano accompanying performances (Tuffiash, 2002, p. 81), calculated the accumulated sight-reading expertise until the age of 18 (Kopiez and Lee, 2008, p. 49) or aggregated the durations of accompaniment and hours of specific sight-reading practice (Meinz and Hambrick, 2010, p. 3). Information on the task-specific accumulated practice duration until the age of 18 or 20 years was used in the case of Ericsson et al. (1993, p. 386), Krampe and Ericsson (1996, p. 347), and Kopiez and Lee (2008, p. 49). In the absence of such data, we used the total accumulated practice time (at the time of the data collection) instead (e.g., in the case of Hallam, 1998, p. 124; McPherson, 2005, author contacted for data; Jabusch et al., 2007, p. 366; and Kopiez et al., 2012, p. 372).

In addition to the predictor variable, the measurement of the outcome variable should be representative of the investigated skill (Ericsson, 2014). Consequently, inter-onset evenness in scale-playing as well as performed (rehearsed) music were identified as truly domain-specific tasks of musical achievement in our sample of studies on music performance. Here, participants' performances were measured either by a reliable psychological evaluation based on psychometric scale construction (e.g., Kornicke, 1992) or by an objective, computer-based, physical measurement such as obtaining the number of correctly performed notes (e.g., Lehmann and Ericsson, 1996) or identifying the inter-onset evenness of scale-playing (e.g., Ericsson et al., 1993; Krampe and Ericsson, 1996; Jabusch et al., 2007). In the case of multiple tasks, as was the case in Ericsson et al. (1993, p. 386) as well as in Krampe and Ericsson (1996, p. 347), we decided to choose the task with the stronger measurement reliability, the highest difficulty and the highest discrimination ability for musical achievement (different movements with each hand (Ericsson et al., 1993, p. 386), simultaneously [Exp. 1], see Krampe and Ericsson, 1996).

### Reliability of identified predictors and outcome variables

For the purpose of adjusting the correlation coefficient of the observed studies for attenuation, the measurement error in the predictor as well as in the outcome variable had to be identified (Hunter and Schmidt, 2004, p. 41). As shown in Table 3, only a small number of studies reported information on the reliability for either the predictor or the outcome variable. Specifically, only Tuffiash (2002, p. 36) reported test-retest reliability in cumulative piano accompaniment performance (*r_xx_* = 0.91) for the quantification of measurement error in the predictor variable. His test-retest reliability estimations were similar to those reported in Bengtsson et al. (2005, p. 1148), who stated a mean test-retest reliability *r_xx_* = 0.89 for the estimation of accumulated deliberate practice obtained from retrospective interviews. Thus, when no reliability was reported for the predictor variable, we used the mean correlation of test-retest reliability according to Bengtsson et al. (2005) to estimate the imperfection of the predictor variable.

**Table 3:** **Reported effect size data on the relationship between indicators of deliberate practice and objective measurement of musical achievement**.

**ID**	**Study design**			**Effect size data**				
	**Sample**	**Predictor**	**Performance measure**	**Sig. report**			Reliability^*^	
				***n***	***r***	***p***	***r*_*xx*_**	***r*_*yy*_**
Kornicke, 1992	College level pianists	Composite number of pieces sight-read	Expert rating of sight-reading performance	73	0.50			0.99
Ericsson et al., 1993—study II	University music majors (pianists)	Accumulated practice	Evenness of inter-onset intervals	24	−0.857	<0.01		
Lehmann and Ericsson, 1996	University music students	Accompanying score	Number of correctly performed notes	16	0.72	<0.01		
Krampe and Ericsson, 1996—study I^+^	Beginning to professional pianists	Accumulated practice (until age of 20)	Evenness of inter-onset intervals	48	−0.62	<0.01		0.97
Hallam, 1998	Beginners	Accumulated practice time	Associated board of the royal schools music (ABRSM)	109	0.67	<0.01		
Meinz, 2000	Beginning to advanced pianists	Number of accompanying performances	Expert rating of sight-reading performance	107	0.57	<0.01		
Tuffiash, 2002	Undergraduate music and non-music majors	Cumulative piano accompaniment performances	Expert ratings of music performances	135	0.426	<0.01	0.91	0.75
McPherson, 2005	Beginners	Accumulated practice time (over 3 years)	Expert rating of performed rehearsed music	99	0.568	<0.01		0.92
Jabusch et al., 2007^+^	School-aged children	Accumulated practice time	Evenness of inter-onset intervals	30	−0.46	<0.05		
Kopiez and Lee, 2008	Piano major students and graduates	Accumulated sight-reading expertise (until age of 18)	Sight-reading achievement	52	0.359	<0.01		
Jabusch et al., 2009^+^^◦◦^	University music students	Life-time deliberate practice	Evenness of inter-onset intervals	19	−0.44	<0.01		
Meinz and Hambrick, 2010^◦◦◦^	Beginners to advanced pianists	Accumulated accompaniments and hours of deliberate sight-reading practice	Expert rating of sight-reading performance	57	0.56	<0.01		0.99
Kopiez et al., 2012—Study II^+^^◦^	University music students (piano major)	Accumulated practice time	Evenness of inter-onset intervals	19	−0.42	<0.05		

+ *Absolute values were used in meta-analysis*.

◦ *Aggregated correlation based on all four correlations between accumulated deliberate practice and outcome variable*.

◦◦ *Aggregated correlation based on two reported correlations between accumulated life-time deliberate practice and outcome variable*.

◦◦◦ *According to Lehmann and Ericsson (1996) the mean correlation of accompaniments (r = 0.63) and hours of deliberate sight-reading practice (r = 0.48) was used as task-specific predictor for sight-reading performance*.

* *Reliability coefficients reported in studies; assumed reliability (if not reported) of predictor variable used for attenuation correction in meta-analysis: r_xx_ = 0.89; assumed reliability (if not reported) of outcome variable (r_yy_) for attenuation correction in meta-analysis: Ericsson et al., 1993 (r_yy_ = 0.91), Lehmann and Ericsson, 1996 (r_yy_ = 0.88), Hallam, 1998 (r_yy_ = 0.91), Meinz, 2000 (r_yy_ = 0.96), Jabusch et al., 2007 (r_yy_ = 0.91), Kopiez and Lee, 2008 (r_yy_ = 0.88), Jabusch et al., 2009 (r_yy_ = 0.91), Kopiez et al., 2012 (r_yy_ = 0.91)*.

To quantify measurement error in the outcome variable, we used the Cronbach's alpha reported in Kornicke (1992, p. 109) for the inter-rater reliability of the sight-reading test and in McPherson (2005, p. 13) for performing rehearsed music. In Krampe and Ericsson (1996, p. 339) and Meinz and Hambrick (2010, p. 4), Cronbach's alpha of the construct reliability for the psychometric measurements could be copied from the respective papers. Finally, in the case of Tuffiash (2002, p. 28) we computed a mean correlation on the basis of all the test-retest reliabilities of sight-reading tests the author reported. For studies in which no measurement error was stated for the outcome variable, we estimated the reliability of the outcome variable's measurement: To estimate the reliability of experts' performance ratings for the outcome variable in Lehmann and Ericsson (1996) and Kopiez and Lee (2008), we used the intercorrelations between the expert judgment of overall impression and the amount of correctly played notes (*r_yy_* = 0.88) as reported in Lehmann and Ericsson (1993, p. 190). In the cases of Ericsson et al. (1993), Jabusch et al. (2007, 2009) and Kopiez et al. (2012), we estimated *r_yy_* = 0.91 as the construct reliability according to Spector et al. (in revision); they computed a mean correlation of test-retest reliability for Jabusch et al.'s (2004) measurement of note-evenness in scale playing. The same test-retest reliability of the scale-analysis by Spector et al. (in revision) was used for the estimation of the test-retest reliability for the ABRSM in Hallam (1998). Along the lines of Bergee (2003), we underestimated the disattenuated correlation by using *r_yy_* = 0.91 and obtained a more conservative correction. Finally, a reliability estimate of *r_yy_* = 0.96 for Meinz (2000) was communicated by the author and also reported in Hambrick et al. (2014, p. 6). In summary, all studies showed a weak attenuation with a 1–17% downwards bias (see Table 4, column A).

**Table 4:** **Statistical values of the meta-analysis**.

**ID**	***N***	***r*_*o*_**	**Reliability**		***A***	***Var(e_*o*_)***	***Var(e_c_*)**	***w***	**Weight [%]**	***r*_*c*_**
			***r*_*xx*_**	***r*_*yy*_**						
Kornicke, 1992	73	0.50	0.89	0.99	0.94	0.01	0.01	64.32	10.10	0.53
Ericsson et al., 1993—study II	24	0.86	0.89	0.91	0.90	0.02	0.03	19.44	3.05	0.96
Lehmann and Ericsson, 1996	16	0.72	0.89	0.88	0.88	0.03	0.04	12.53	1.97	0.81
Krampe and Ericsson, 1996—study I	48	0.62	0.89	0.97	0.93	0.01	0.01	41.44	6.51	0.67
Hallam, 1998	109	0.67	0.89	0.91	0.90	0.00	0.01	88.28	13.87	0.74
Meinz, 2000	107	0.57	0.89	0.96	0.92	0.00	0.01	91.42	14.36	0.62
Tuffiash, 2002	135	0.43	0.91	0.75	0.83	0.00	0.01	92.14	14.47	0.52
McPherson, 2005	99	0.57	0.89	0.92	0.90	0.01	0.01	81.06	12.73	0.63
Jabusch et al., 2007	30	0.46	0.89	0.91	0.90	0.02	0.02	24.30	3.82	0.51
Kopiez and Lee, 2008	52	0.36	0.89	0.88	0.88	0.01	0.01	40.73	6.40	0.41
Jabusch et al., 2009	19	0.44	0.89	0.91	0.90	0.03	0.03	15.39	2.42	0.49
Meinz and Hambrick, 2010	57	0.56	0.89	0.99	0.94	0.01	0.01	50.22	7.89	0.60
Kopiez et al., 2012—study II	19	0.42	0.89	0.91	0.90	0.03	0.03	15.39	2.42	0.47

*N, sample size; r_o_, observed correlation (Hunter and Schmidt, 2004, p. 96); r_xx_, reliability of predictor variable (error of measurement in the predictor variable, Hunter and Schmidt, 2004, p. 96); r_yy_, reliability of outcome variable (error of measurement in the outcome variable, Hunter and Schmidt, 2004, p. 96); A, attenuation factor (r_o_/r_c_, Hunter and Schmidt, 2004, p. 118); Var(e_o_), sampling error variance of each study's uncorrected correlation (Hunter and Schmidt, 2004, p. 87); Var(e_c_), sampling error variance of each study's corrected correlation (Hunter and Schmidt, 2004, p. 119); w, study weight (Hunter and Schmidt, 2004, p. 125); r_c_, corrected study correlation (Hunter and Schmidt, 2004, p. 118); weighted mean observed correlation r_o_ = 0.54 (Hunter and Schmidt, 2004, p. 81); frequency-weighted average squared error S^2^_r_ = 0.01 (Hunter and Schmidt, 2004, p. 81); mean true score correlation ρ = 0.61 (Hunter and Schmidt, 2004, p. 125); variance of true score correlations S^2^_ρ_ = 0 (Hunter and Schmidt, 2004, p. 126); observed variance of the corrected correlations S^2^_r_c__ = 0.01 (Hunter and Schmidt, 2004, p. 126); variance in corrected correlations attributable to all artifacts S^2^_e_c__ = 0.01 (Hunter and Schmidt, 2004, p. 126); complete variance in corrected correlations (81.2%) is attributable to all artifacts (Hunter and Schmidt, 2004, p. 401); Q-test on study homogeneity as well as I^2^ suggest no significant variation across studies (I^2^ = 0.00; Q(12) = 8.19, p = 0.77)*.

### Statistical reanalysis and meta-analysis with correlations corrected for artifacts

All studies reported correlations that could be used for quantifying the effect of deliberate practice on the musical achievement (see Table 3). Meinz and Hambrick (2010) reported multiple predictors of sight-reading skill along the theoretical outline for the acquisition of sight-reading skill (Lehmann and Ericsson, 1996; Kopiez and Lee, 2006). We aggregated the two predictors, *number of accompanying events/activities* (*r* = 0.63) and *hours of sight-reading practice* (*r* = 0.48), into a mean correlation (*r* = 0.56) to be used as a global predictor for sight-reading performance (see Table 3). As a result of a 2 × 2 experimental design, four correlations of pianists' accumulated task-specific practice times and scale performances were reported in Kopiez et al. (2012). Again, the four individual correlations (*r_L__i_* = −0.47; *r_L__o_* = −0.23; *r_R__i_* = −0.46; *r_R__o_* = −0.50) were aggregated to the study's effect size (*r* = −0.42) (Kopiez et al., 2012, Table 6 on p. 372; see comment on negative values below). Finally, in the case of Jabusch et al. (2009, p. 77), two correlations between total life-time practice and music performance (as measured by evenness in scale playing on various dates with a distance of 1 year; *r*_1_ = −0.47; *r*_2_ = −0.40) were reported. We calculated and used the mean correlation (|*r*| = 0.44) in our meta-analysis.

Jabusch et al.'s (2004) scale-playing paradigm generally resulted in negative correlations (see Table 3). Since the authors report the median of the scale-related inter-onset interval standard deviation (medSDIOI) as an indicator for evenness, a low medSDIOI signals high evenness. A positive association between accumulated practice times and the medSDIOI can still be postulated: the longer the pianist's deliberate practice durations, the smaller the degree of unevennes. For the sake of simplicity we used the absolute values of the correlations reported in our meta-analysis (this also applies to Ericsson et al., 1993; Krampe and Ericsson, 1996; Jabusch et al., 2007, 2009; Kopiez et al., 2012).

Finally, the observed correlations as well as the reliabilities of predictor and outcome variables were entered into the Hunter-Schmidt Meta-Analysis software (Schmidt and Le, 2005) so that we could correct all observable correlations for artifacts (Hunter and Schmidt, 2004, p. 75) within the meta-analysis and estimate the population correlation for the “true” effect size (see Table 4).

## Results

### Statistical procedure

The observed correlation (*r_o_*) for each study was transformed into its disattenuated *r_c_* value. This disattenuation procedure is based on the assumption that the observed correlation (*r_o_*) comprises the “true” value plus the influence of a measurement error that depends on the reliability of both the predictor (*r_xx_*) and outcome (*r_yy_*) variable. According to Hunter and Schmidt (2004), the *r_o_* value has to be corrected for limited reliability of both variables, and this correction is implemented in the *Hunter-Schmidt Meta-Analysis Programs* (see Schmidt and Le, 2005). Detailed results with all steps and for each study are shown in Table 4. It is remarkable that 81.2% of the complete variance in all corrected correlations was attributable to the artifacts, a finding which leaves no residual variance to be explained (for an explanation, see Hunter and Schmidt, 2004, p. 401). In other words, our meta-analysis is based on an homogenous corpus of data (*Q*(12) = 8.19, *p* = 0.77; *I*^2^ = 0.00%) which is the outcome of a careful sampling and study selection, guided by the criteria of task-specific practice and objective measurements of music performance.

### Main outcome

**Figure 2:**
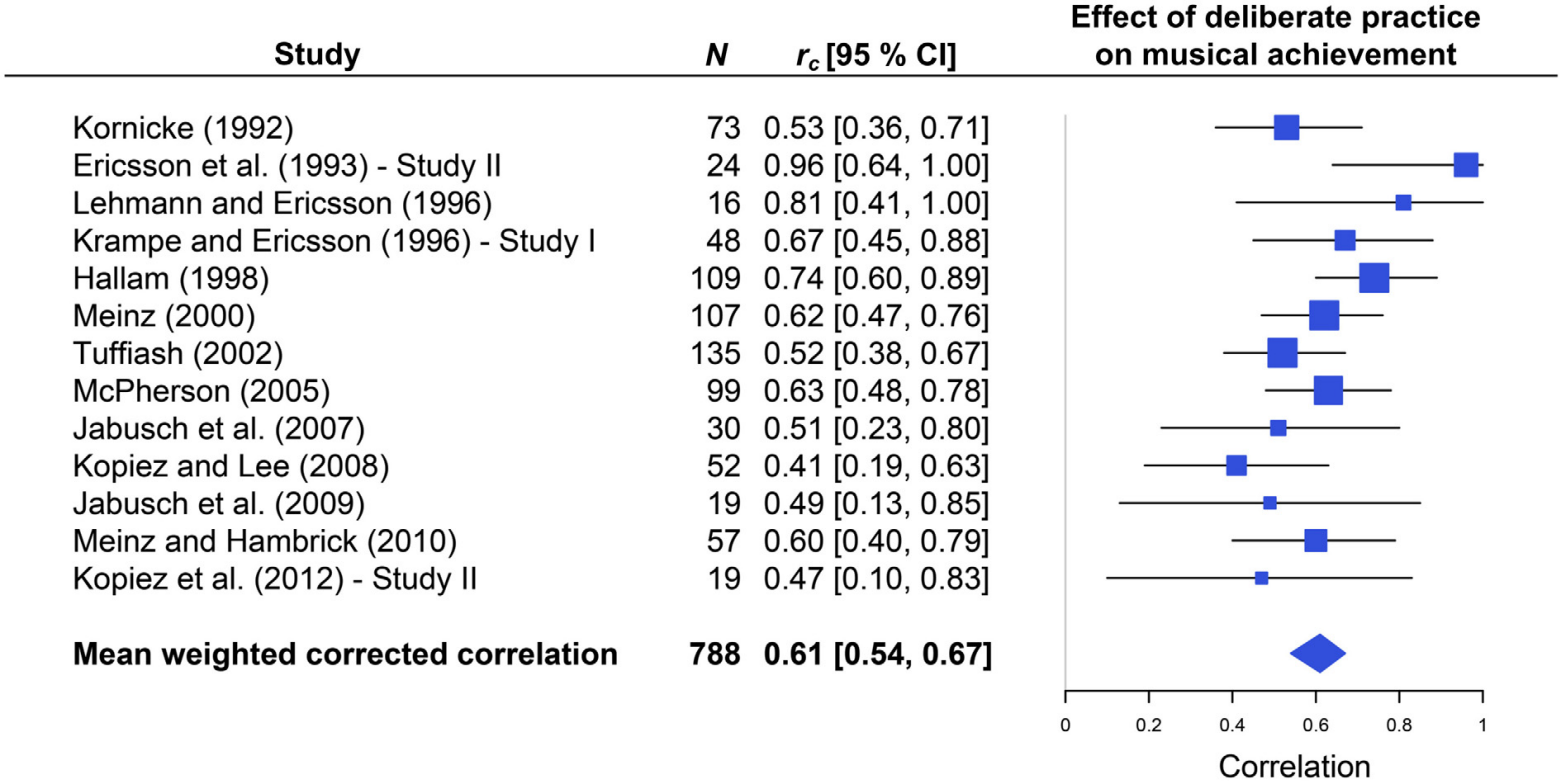
**Forest plot of corrected effect sizes for individual studies and of the aggregated mean effect size (*r_c_* = 0.61, 95% CI [0.54, 0.67]) based on the total number of *N* = 788 participants**. Error bars indicate 95% CI; the size of the squares corresponds to the relative weight of the study.

The result from 13 studies regarding the effect of the indicators of DP on musical achievement is summarized in Figure 2 using a forest plot. Our meta-analysis yielded an average aggregated corrected effect size of *r_c_* = 0.61, with CI 95% [0.54, 0.67]. According to Cohen's benchmarks (1988, p. 80), this corresponds to a large effect. The size of the squares in the forest plot indicates each study's weight and error bars delimit the 95% CI. The remarkably strong relationship between task-specific practice and musical achievement as measured by objective means is only one facet of the aggregated and corrected correlations. Another facet of the results is the 95% CI as a measure of dispersion for the population effect which is rather narrow [0.54, 0.67] and positive. This feature indicates the stability of our finding. The forest plot also shows that the aggregated correlation is not biased by one or two studies with extreme relative weights. Rather, a total of 4 studies (Hallam, 1998; Meinz, 2000; Tuffiash, 2002; McPherson, 2005) with high relative weights contribute 50% to the aggregated result.

### Test for publication bias

**Figure 3:**
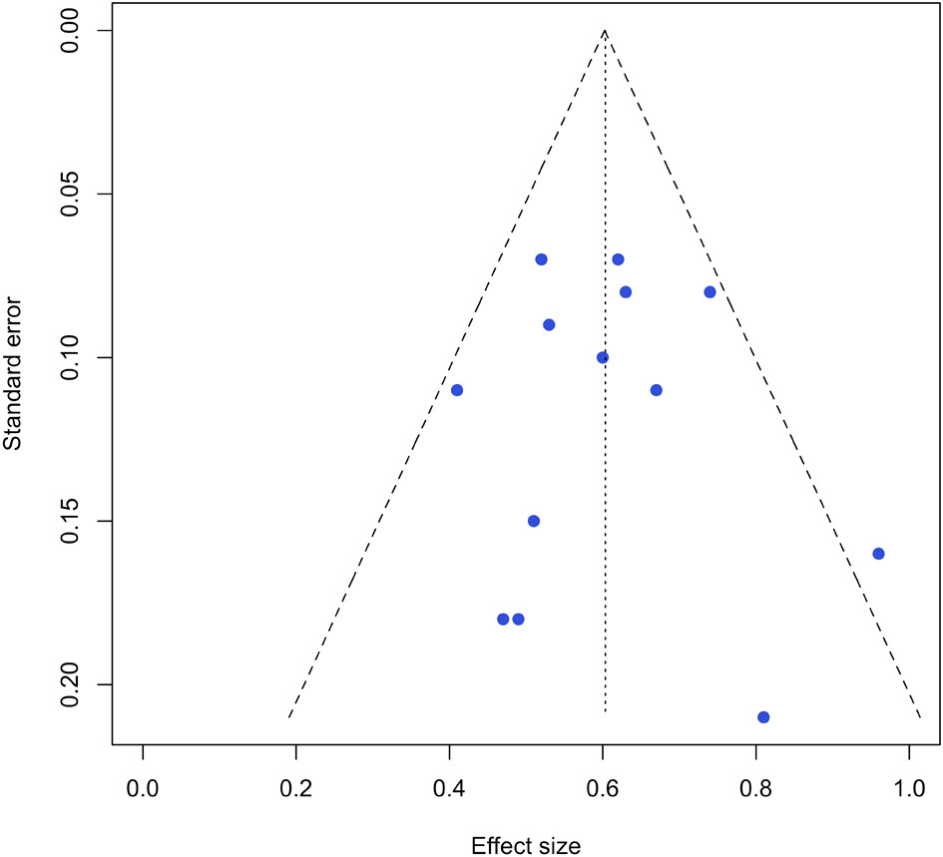
**Funnel plot of studies' effect sizes (*r_c_*) against standard error of effect sizes as a test for publication bias**.

Evidence suggests that due to their selective decision processes and preference for significant results, peer-reviewed journals only partially reflect research activities (Rothstein et al., 2005). This so-called publication or availability bias is an indicator for the existence of unpublished results, and it is a sign of how strongly those unpublished studies could influence the results of a meta-analysis. To detect the presence of a systematic selection bias of publications, we used the so-called funnel plot (Egger et al., 1997) (see Figure 3). If publication bias is present, the distribution of results will form an asymmetrically shaped funnel. Fortunately, Figure 3 shows a nearly symmetrical distribution of effect sizes in relation to the standard error (the indicator of precision). With the exception of one, the effect sizes lie within the funnel's shape and are centered symmetrically around the aggregated mean of *r_c_* = 0.61. Such considerably low bias is one of the strengths of our meta-analysis and the result of carefully defined criteria for inclusion (see Figure 1).

## Discussion

One of the main results of our meta-analysis is the identification of a reliable, aggregated correlation between task-relevant practice and objectively measured musical achievement. Although the central parameter of our analysis of 13 studies is similar to the one calculated by Hambrick et al. (2014) on the basis of 8 studies, there are some marked differences between both approaches. Our results may currently represent the best estimate of this correlation given the published data and methodological tools.

### Comparison of our findings to those by Hambrick et al. (2014)

An important step in the use of correlation coefficients in meta-analyses is the correction for attenuation (Hunter and Schmidt, 2004). It considers the reliability of the outcome and predictor variables in a study. Although we chose conservative estimates of reliability for the disattenuation procedure in the present paper, our resulting correlation value is higher (*r_c_* = 0.61) than Hambrick et al.'s (2014) (*r_c_* = 0.52), and it covers a smaller confidence interval (95% CI [0.54, 0.67]) compared to theirs (95% CI [0.43, 0.64]). Therefore, we conclude that our meta-analysis is a more reliable approximation of the “true” correlation between task-relevant practice (including DP) and musical achievement.

In some instances, the predictors we used were different from those Hambrick et al. (2014) had used for their study. For example, they selected the value of *r_o_* = 0.25 from the sight-reading study by Kopiez and Lee (2008). However, this correlation between task-relevant study (i.e., sight-reading expertise) and actual sight-reading achievement was based on the lifetime accumulated practice time in sight-reading (up to the time of data collection). In line with the criteria for the calculation of accumulated practice time employed in Ericsson et al. (1993); Ericsson et al. (Study II, see Table 3), and for reasons of comparability, we used the correlation between accumulated sight-reading expertise up to the age of 18 years and sight-reading performance (*r_o_* = 0.36; Kopiez and Lee, 2008) for our meta-analysis. Life-time accumulated practice durations were only used when no information on the task-specific accumulated practice time until the age of 18 or 20 years could be obtained from the studies. We believe that the careful selection of studies and variables based on selection criteria of objective measurement for the outcome (performance) variable and clear calculations of accumulated practice durations are the main reasons for the differences between Hambrick et al.'s results and ours.

### The role of possible further moderating variables on performance

The discussion on the influence of variables other than study durations that might influence musical achievement is ongoing and interesting. Here, we wish to comment on the tendency of authors to use headings for publications that can be misleading for the uninformed reader. For example, Meinz and Hambrick (2010) insinuate that there might be (heritable) variables which have a significant influence on musical achievement, and they suggest working memory capacity as such an influential factor. Yet, their main finding regarding the central role of various forms of relevant practice on sight-reading achievement (within a range from *r_o_* = 0.37 to 0.67) implies that working memory capacity can only contribute a smaller proportion of the variance (*r_o_* = 0.28). Although the authors conclude “that deliberate practice accounted for nearly half of the total variance in piano sight-reading performance” (Meinz and Hambrick, 2010, p. 914), the article title, “Limits on the Predictive Power of Domain-Specific Experience and Knowledge in Skilled Performance,” defames the role of deliberate practice. A second case is the publication by Ruthsatz et al. (2008) in which the authors found a low correlation between general intelligence (IQ) and musical achievement of *r_o_* = 0.25 (Study 1), 0.11 (Study 2A), and −0.01 (Study 2B) but a large one between accumulated practice time and musical achievement (*r_o_* = 0.34 [Study 1], 0.31 [Study 2A], and 0.54 [Study 2B]). Their combination of “other” variables exceeds the influence of deliberate practice times only when the aggregated correlations of IQ and music audiation are compared with the influence of the individual predictor of practice. However, it is well-known that Gordon's tests of audiation (AMMA), which Ruthsatz uses, is influenced by musical experience and thus already captures effects of DP. In light of such findings, the authors' claim that “higher-level musicians report significantly higher mean levels of characteristics such as general intelligence and music audiation, in addition to higher levels of accumulated practice time” (Ruthsatz et al., 2008, p. 330) is grossly misleading.

**Figure 4:**
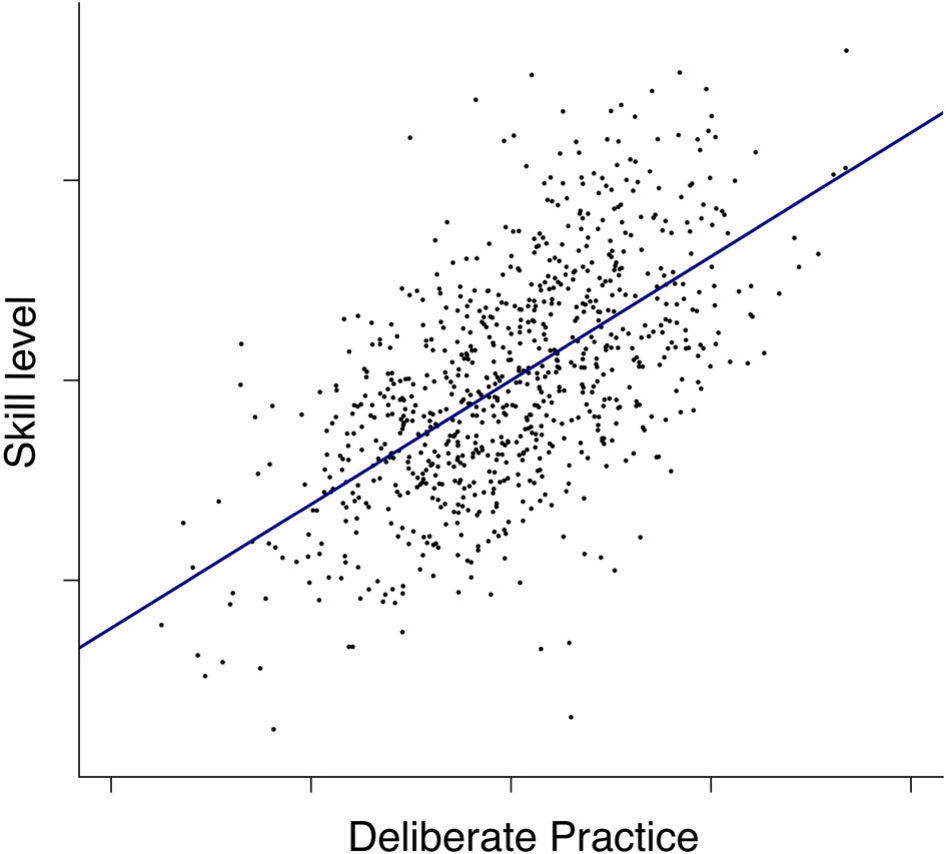
**Illustration of the (linear) correlation (*r_c_* = 0.61) between indicators of DP and musical achievement based on a simulation with *N* = 788 normal distributed cases with a mean of 0**. An increase of 1 unit on the *x*-axis corresponds to an increase of 0.61 units on the *y*-axis.

Another argument for a differentiated view of our findings arises from the erroneous interpretation of *r* (or *r_c_*) values as *r^2^* values known from common variance. For example, Hambrick et al. (2014, p. 7) state: “On average across studies, deliberate practice explained about 30% of the reliable variance in music performance.” However, according to Hunter and Schmidt (2004, p. 190), this is a problematic interpretation with regard to findings from a meta-analysis, because the *r^2^* value is “related only in a very nonlinear way to the magnitudes of effect sizes that determine their impact in the real world.” Instead, relationships between variables should be interpreted in terms of linear relationships. Therefore, we could illustrate the relevance of our meta-analytical finding by means of a correlation simulation based on a sample size of *N* = 788 and a given correlation of *r_c_* = 0.61. Figure 4 displays this simulation with the linear increase of one unit on the *x*-axis corresponding to an increase of musical skill level or achievement by 0.61 units. If we expressed this in terms of an experimental between-groups design, this *r_c_* value of 0.61 would translate to a Cohen's *d* of 1.52 which implicates a very large effect (Ellis, 2010, p. 16). In our view, this is a strong argument for the eminent importance of long-term DP for skill acquisition and achievement.

In summary, it is incorrect to interpret our findings (*r_c_* = 0.61) as evidence that DP explains 36% of the variance in attained music performance. Instead, it is correct to state that the currently trackable correlation between an approximation of deliberate practice with indicators such as solitary study or task-relevent training experiences is related to measurements of music performance with *r_c_* = 0.61.

## Future perspectives

Currently, there is a lack of controlled empirical studies based on the expertise theory in the domain of music. This problem is reflected in the small number of studies (*N* = 13) conducted over the last 20 years which matched the rigorous selection criteria of our meta-analysis. One of the main challenges in the future will therefore be to extend the base of reliable experimental data. This means that studies should use state of the art measurements of relevant deliberate practice durations (e.g., year-by-year retrospective reports, diaries etc.) and objective and reliable assessments of performance variables (e.g., preferably hard performance measurements or consensual expert ratings of performance achievements). All of this was demanded many years ago (e.g., Ericsson and Smith, 1991). The use of standardized performance tasks (e.g., intact performance such as sight-reading with a pacing voice or isolated subskills such as scale playing at a given speed) with the objective measurement of performance and additional information on their reliabilities will be mandatory for investigating the “true” relationship between task-specific practice and musical achievement. This demand underscores Ericsson's (2014, p. 16) claim that “the expert-performance framework restricts its research to objectively measurable performance. It rejects research based on supervisor ratings and other social indicators….” Consequently, self-reports on abilities, the rating of a musican's skill level by an orchestra's conductor, and reports of parents about their child's level of achievement are not acceptable as objective indicators of performance. The question of whether the expert performance framework generalizes to the general population also awaits investigation (Ericsson, 2014). As our findings are currently limited to music, it will be necessary to cross-validate them with meta-analytic findings in other domains of expertise, such as sports or chess. The likelihood of their being generalizable is high, though, due to the methodological rigor of our study.

One general problem for the domain of music is that time estimations of practice durations are only approximate indicators of deliberate practice, which by definition only constitutes optimized practice and training activities. If we were able to identify the actual amount of deliberate practice inherent in the durational estimates that currently also include suboptimal practice activities, especially in sub-expert populations, then the aggregated correlations could certainly be higher than *r_c_* = 0.61. Solitary practice might also not cover all aspects of deliberate practice (e.g., competition experience). Thus, our figure of *r_c_* = 0.61 might currently be considered as the theoretically lower bound of the true effect of DP. The most suitable future studies that could untangle this empirical conundrum would include micro-analyses of practice activities and in particular longitudinal studies like the one's by McPherson et al. (2012) for music; or Gruber et al. (1994) for chess. Such studies should be the natural next step in the quest for the factors that mediate expert and exceptional performance.

## Author contributions

Conceived and designed the meta-analysis: Andreas C. Lehmann, Reinhard Kopiez, Friedrich Platz, Anna Wolf. Conducted the search for references: Reinhard Kopiez, Anna Wolf, Friedrich Platz, Andreas C. Lehmann. Analyzed the data: Friedrich Platz, Anna Wolf, Reinhard Kopiez, Andreas C. Lehmann. Wrote the paper: Friedrich Platz, Reinhard Kopiez, Andreas C. Lehmann, Anna Wolf.

### Conflict of interest statement

The authors declare that the research was conducted in the absence of any commercial or financial relationships that could be construed as a potential conflict of interest.

## References

[B1] Bengtsson S. L. Nagy Z. Skare S. Forsman L. Forssberg H. Ullén F. (2005). Extensive piano practicing has regionally specific effects on white matter development. Nat. Neurosci. 8, 1148–1150 10.1038/nn1516 16116456

[B2] Bergee M. J. (2003). Faculty interjudge reliability of music performance evaluation. J. Res. Music Educ. 51, 137–150 10.2307/3345847

[B3] Booth A. Papaioannou D. Sutton A. (2012). Systematic Approaches to a Successful Literature Review. London: Sage

[B5] Borenstein M. (2010). Comprehensive Meta-Analysis (2.0) [Computer software]. Englewood, NJ: Biostat

[B6] Brandler S. Rammsayer T. H. (2003). Differences in mental abilities between musicians and non-musicians. Psychol. Music 31, 123–138 10.1177/0305735603031002290

[B7] Campitelli G. Gobet F. (2011). Deliberate practice: necessary but not sufficient. Curr. Dir. Psychol. Sci. 20, 280–285 10.1177/0963721411421922

[B8] Chabris C. F. (1999). Prelude or requiem for the “Mozart effect?” Nature 400, 826–827 10.1038/23608 10476958

[B9] Cohen J. (1988). Statistical Power Analysis for the Behavioral Sciences. Hillsdale, NJ: Lawrence Erlbaum

[B10] Cohn L. D. Becker B. J. (2003). How meta-analysis increases statistical power. Psychol. Methods 8, 243–253 10.1037/1082-989X.8.3.243 14596489

[B11] Cooper H. Hedges L. V. Valentine J. C. (eds.). (2009). The Handbook of Research Synthesis and Meta-Analysis. New York, NY: Russell Sage Foundation

[B12] Deeks J. J. Higgins J. P. T. Altman D. G. (2008). Analysing data and undertaking meta-analyses, in Cochrane Handbook for Systematic Reviews of Interventions, eds Higgins J. P. T. Green S. (Chichester: Wiley-Blackwell), 243–296 10.1002/9780470712184.ch9

[B13] Detterman D. K. (ed.) (2014). Acquiring expertise: ability, practice, and other influences [Special issue]. Intelligence 45, 1–123

[B14] Egger M. Smith G. D. Schneider M. Minder C. (1997). Bias in meta-analysis detected by a simple, graphical test. Br. Med. J. 315, 629–634 10.1136/bmj.315.7109.629 9310563 PMC2127453

[B15] Ellis P. D. (2010). The Essential Guide to Effect Sizes: Statistical Power, Meta-Analysis, and the Interpretation of Research Results. Cambridge: Cambridge University Press 10.1017/CBO9780511761676

[B16] Ericsson K. A. (ed.). (1996). The Road to Excellence: the Acquisition of Expert Performance in the Arts and Sciences, Sports and Games. Mahwah, NJ: Lawrence Erlbaum

[B17] Ericsson K. A. (2006). The influence of experience and deliberate practice on the development of superior expert performance, in The Cambridge Handbook of Expertise and Expert Performance, eds Ericsson K. A. Charness N. Feltovich P. J. Hoffman R. R. (Cambridge: Cambridge University Press), 683–703

[B18] Ericsson K. A. (2014). Why expert performance is special and cannot be extrapolated from studies of performance in the general population: A response to criticisms. Intelligence 45, 81–103 10.1016/j.intell.2013.12.001

[B19] ^*^ Ericsson K. A. Krampe R. T. Tesch-Römer C. (1993). The role of deliberate practice in the acquisition of expert performance. Psychol. Rev. 100, 363–406 10.1037/0033-295X.100.3.363

[B20] Ericsson K. A. Lehmann A. C. (1999). Expertise, in Encyclopedia of Creativity, eds Runco M. A. Pritzker S. (New York, NY: Academic Press), 695–707

[B21] Ericsson K. A. Smith J. (1991). Prospects and limits in the empirical study of expertise: an introduction, in Toward a General Theory of Expertise: Prospects and Limits, eds Ericsson K. A. Smith J. (Cambridge: Cambridge University Press), 1–38

[B22] Gruber H. Lehmann A. C. (2008). Entwicklung von Expertise und Hochleistung in Musik und Sport [The development of expertise and superior performance in music and sports], in Angewandte Entwicklungspsychologie (Enzyklopädie der Psychologie, Vol. C/V/7), eds Petermann F. Schneider W. (Göttingen: Hogrefe), 497–519

[B23] Gruber H. Renkl A. Schneider W. (1994). Expertise und Gedächtnisentwicklung: längsschnittliche Befunde aus der Domäne Schach [expertise and the development of memory: longitudinal results from the domain of chess]. Zeitschrift für Entwicklungspsychologie und Pädagogische Psychologie 26, 53–70

[B24] Gulliksen H. (1950). Theory of Mental Tests. New York, NY: Wiley and Sons 10.1037/13240-000

[B25] ^*^ Hallam S. (1998). The predictors of achievement and dropout in instrumental tuition. Psychol. Music 26, 116–132 10.1177/0305735698262002

[B26] Hambrick D. Z. Meinz E. J. (2011). Limits on the predictive power of domain-specific experience and knowledge in skilled performance. Curr. Dir. Psychol. Sci. 20, 275–279 10.1177/0963721411422061

[B27] Hambrick D. Z. Oswald F. L. Altmann E. M. Meinz E. J. Gobet F. Campitelli G. (2014). Deliberate practice: is that all it takes to become an expert? Intelligence 45, 34–45 10.1016/j.intell.2013.04.001

[B28] Hetland L. (2000). Listening to music enhances spatial-temporal reasoning: evidence for the “Mozart effect.” J. Aesthet. Educ. 34, 105–148 10.2307/3333640

[B29] Higgins J. P. T. Green S. (eds.). (2008). Cochrane Handbook for Systematic Reviews of Interventions. Chichester: Wiley-Blackwell 10.1002/9780470712184

[B30] Howe M. J. A. Davidson J. W. Sloboda J. A. (1998). Innate talents: reality or myths? Behav. Brain Sci. 21, 399–442 10.1017/S0140525X9800123X 10097018

[B31] Hunter J. E. Schmidt F. L. (2004). Methods of Meta-Analysis: Correcting Error and Bias in Research Findings. London: Sage

[B32] ^*^ Jabusch H.-C. Alpers H. Kopiez R. Vauth H. Altenmüller E. (2009). The influence of practice on the development of motor skills in pianists: a longitudinal study in a selected motor task. Hum. Mov. Sci. 28, 74–84 10.1016/j.humov.2008.08.001 18845349

[B33] Jabusch H.-C. Vauth H. Altenmüller E. (2004). Quantification of focal dystonia in pianists using scale analysis. Mov. Disord. 19, 171–180 10.1002/mds.10671 14978672

[B34] ^*^ Jabusch H.-C. Young R. Altenmüller E. (2007). Biographical predictors of music-related motor skills in children pianists, in International Symposium on Performance Science, eds Williamon A. Coimbra D. (Porto: Association Europeìenne des Conservatoires, Acadeìmies de Musique et Musikhochschulen (AEC)), 363–368

[B35] Kämpfe J. Sedlmeier P. Renkewitz F. (2011). The impact of background music on adult listeners: a meta-analysis. Psychol. Music 39, 424–448 10.1177/0305735610376261

[B36] Kopiez R. (2012). The role of replication studies and meta-analyses in the search of verified knowledge, in 12th International Conference on Music Perception and Cognition (ICMPC), 23–28 July, eds Cambouropoulos E. Tsougras C. Mavromatis P. Pastiadis K. (Thessaloniki: Aristotle University of Thessaloniki), 64–65

[B37] Kopiez R. Galley N. Lee J. I. (2006). The advantage of being non-right-handed: the influence of laterality on a selected musical skill (sight reading achievement). Neuropsychologia 44, 1079–1087 10.1016/j.neuropsychologia.2005.10.023 16321405

[B38] Kopiez R. Galley N. Lehmann A. C. (2010). The relation between lateralisation, early start of training, and amount of practice in musicians: a contribution to the problem of handedness classification. Laterality 15, 385–414 10.1080/13576500902885975 19462271

[B39] ^*^ Kopiez R. Jabusch H.-C. Galley N. Homann J.-C. Lehmann A. C. Altenmüller E. (2012). No disadvantage for left-handed musicians: the relationship between handedness, felt constraints and performance-related skills in pianists and string players. Psychol. Music 40, 357–384 10.1177/0305735610394708

[B40] Kopiez R. Lee J. I. (2006). Towards a dynamic model of skills involved in sight reading music. Music Educ. Res. 8, 97–120 10.1080/14613800600570785

[B41] ^*^ Kopiez R. Lee J. I. (2008). Towards a general model of skills involved in sight reading music. Music Educ. Res. 10, 41–62 10.1080/14613800701871363

[B42] ^*^ Kornicke L. E. (1992). An Exploratory Study of Individual Difference Variables in Piano Sight-Reading Achievement. PhD, Indiana University

[B43] Krampe R. T. Charness N. (2006). Aging and expertise, in Cambridge Handbook of Expertise and Expert Performance, eds Ericsson A. K. Charness N. Feltovich P. J. Hoffman R. R. (Cambridge: Cambridge University Press), 723–742 10.1017/CBO9780511816796.040

[B44] ^*^ Krampe R. T. Ericsson K. A. (1996). Maintaining excellence: deliberate practice and elite performance in young and older pianists. J. Exp. Psychol. Gen. 125, 331–359 10.1037/0096-3445.125.4.331 8945787

[B45] Lehmann A. C. (1997). The acquisition of expertise in music: efficiency of deliberate practice as a moderating variable in accounting for sub-expert performance, in Perception and Cognition of Music, eds Deliège I. Sloboda J. A. (Hove: Psychology Press), 161–187

[B46] Lehmann A. C. (2005). Musikalischer Fertigkeitserwerb (Expertisierung): Theorie und Befunde [musical skill acquisition (expertization): theories and results], in Musikpsychologie (Handbuch der Systematischen Musikwissenschaft, Vol. 3, eds De La Motte-Haber H. Rötter G. (Laaber: Laaber), 568–599

[B47] Lehmann A. C. Ericsson K. A. (1993). Sight-reading ability of expert pianists in the context of piano accompanying. Psychomusicology 12, 182–195 10.1037/h0094108

[B48] ^*^ Lehmann A. C. Ericsson K. A. (1996). Performance without preparation: Structure and acquisition of expert sight-reading and accompanying performance. Psychomusicology 15, 1–29 10.1037/h0094082

[B49] Lehmann A. C. Gruber H. (2006). Music, in Cambridge Handbook on Expertise and Expert Performance, eds Ericsson K. A. Charness N. Feltovich P. Hoffman R. R. (Cambridge: Cambridge University Press), 457–470 10.1017/CBO9780511816796.026

[B50] Masicampo E. J. Lalande D. R. (2012). A peculiar prevalence of p values just below.05. Q. J. Exp. Psychol. 65, 2271–2279 10.1080/17470218.2012.711335 22853650

[B51] ^*^ McPherson G. E. (2005). From child to musician: skill development during the beginning stages of learning an instrument. Psychol. Music 33, 5–35 10.1177/0305735605048012

[B52] McPherson G. E. Davidson J. W. Faulkner R. (2012). Music in Our Lives: Rethinking Musical Ability, Development and Identity. Oxford: Oxford University Press 10.1093/acprof:oso/9780199579297.001.0001

[B53] ^*^ Meinz E. J. (2000). Experience-based attenuation of age-related differences in music cognition tasks. Psychol. Aging 15, 297–312 10.1037/0882-77974.15.2.297 10879584

[B54] ^*^ Meinz E. J. Hambrick D. Z. (2010). Deliberate practice is necessary but not sufficient to explain individual differences in piano sight-reading skill: the role of working memory capacity. Psychol. Sci. 21, 914–919 10.1177/0956797610373933 20534780

[B55] Mishra J. (2014). Improving sightreading accuracy: a meta-analysis. Psychol. Music 42, 131–156 10.1177/0305735612463770

[B56] Nandagopal K. Ericsson K. A. (2012). Enhancing students' performance in traditional education: Implications from the expert performance approach and deliberate practice, in APA Educational psychology handbook (Vol. 1, Theories, constructs, and critical issues), eds Harris K. R. Graham S. Urdan U. (Washington, DC: American Psychological Association), 257–293

[B57] Pietschnig J. Voracek M. Formann A. K. (2010). Mozart effect–Shmozart effect: a meta-analysis. Intelligence 38, 314–323 10.1016/j.intell.2010.03.00110476958

[B58] Platz F. Kopiez R. (2012). When the eye listens: a meta-analysis of how audio-visual presentation enhances the appreciation of music performance. Music Percept. 30, 71–83 10.1525/mp.2012.30.1.71

[B59] Rosenthal R. (1979). The “file drawer problem” and tolerance for null results. Psychol. Bull. 86, 638–641 10.1037/0033-2909.86.3.638

[B60] Rothstein H. R. Hopewell S. (2009). Grey literature, in The Handbook of Research Synthesis and Meta-Analysis, 2nd Edn., eds Cooper H. Hedges L. V. Valentine J. C. (New York, NY: Russel Sage Foundation), 103–125

[B61] Rothstein H. R. Sutton A. J. Borenstein M. (eds.). (2005). Publication Bias in Meta-Analysis: Prevention, Assessment and Adjustments. Chichester: John Wiley & Sons 10.1002/0470870168

[B62] Ruthsatz J. Detterman D. Griscom W. S. Cirullo B. A. (2008). Becoming an expert in the musical domain: it takes more than just practice. Intelligence 36, 330–338 10.1016/j.intell.2007.08.003

[B63] Schmidt F. L. Le H. A. (2005). Hunter-Schmidt Meta-Analysis Programs. V 1.1. Iowa, IA

[B64] Shavinina L. (2009). A unique type of representation is the essence of giftedness: towards a cognitive-developmental theory, in International Handbook on Giftedness, ed Shavinina L. (Berlin: Springer Science and Business Media), 231–257

[B65] Sloboda J. A. (2000). Individual differences in music performance. Trends Cogn. Sci. 4, 397–403 10.1016/S1364-6613(00)01531-X 11025283

[B67] ^*^ Tuffiash M. (2002). Predicting Individual Differences in Piano Sight-Reading Skill: Practice, Performance, and Instruction. Unpublished master's thesis, Florida State University

[B68] Ullén F. Forsman L. Blom O. Karabanov A. Madison G. (2008). Intelligence and variability in a simple timing task share neural substrates in the prefrontal white matter. J. Neurosci. 28, 4238–4243 10.1523/JNEUROSCI.0825-08.2008 18417703 PMC6670305

[B69] Vandervert L. R. (2009). Working memory, the cognitive functions of the cerebellum and the child prodigy, in International Handbook on Giftedness, ed Shavinina L. (Berlin: Springer Science and Business Media), 295–316

[B70] Winner E. (1996). The rage to master: the decisive role of talent in the visual arts, in The Road to Excellence: the Acquisition of Expert Performance in the Arts and Sciences, Sports and Games, ed Ericsson K. A. (Mahwah, NJ: Lawrence Erlbaum), 271–301

[B71] Ziegler A. (2009). Research on giftedness in the 21st century, in International Handbook on Giftedness, ed Shavinina L. (Berlin: Springer Science and Business Media), 1509–1524 10.1007/978-1-4020-6162-2_78

